# Effect of Osteopathic Manipulation in an Autism Spectrum Child With Speech Impairment and Attention Deficit: A Case Report

**DOI:** 10.7759/cureus.56809

**Published:** 2024-03-24

**Authors:** H V Sharath, Raghumahanti Raghuveer, Moh'd Irshad Qureshi, Pratiksha A Warghat, Sakshi Desai, Neha A Brahmane

**Affiliations:** 1 Department of Paediatric Physiotherapy, Ravi Nair Physiotherapy College, Datta Meghe Institute of Higher Education and Research, Wardha, IND; 2 Department of Neurophysiotherapy, Ravi Nair Physiotherapy College, Datta Meghe Institute of Higher Education and Research, Wardha, IND

**Keywords:** manual therapy, cranio-sacral osteopathy, visceral manipulation, pediatric osteopathy, autism spectrum disorder and anxiety disorder

## Abstract

Autism spectrum disorder (ASD) is a complex neurodevelopmental condition characterized by challenges in communication, social interaction, and repetitive behaviors. Children with ASD often experience comorbidities such as speech impairment and attention deficit, which can significantly impact their quality of life and ability to engage in daily activities. This case report aims to investigate the potential benefits of osteopathic manipulation in addressing speech impairment and attention deficit in a child diagnosed with ASD. A four-year-old male child diagnosed with ASD, presenting with speech impairment and attention deficit, received a series of osteopathic manipulation sessions over a period of 12 weeks. The treatment protocol was tailored to address musculoskeletal dysfunctions, cranial restrictions, somatic dysfunctions, and digestive system dysfunctions identified through osteopathic assessment. Following the osteopathic manipulation sessions, improvements were observed in the child’s speech fluency and attention span. The child demonstrated increased engagement in communication activities and showed enhanced focus during therapy sessions. Additionally, improvements were noted in the child’s overall behavior and social interaction skills. This case report suggests that osteopathic manipulation may be a beneficial adjunctive therapy for children with ASD experiencing speech impairment and attention deficit. Further research with larger sample sizes and controlled study designs is warranted to validate these findings and elucidate the mechanisms underlying the observed improvements. Osteopathic manipulation holds promise as a non-invasive, holistic approach to addressing various aspects of ASD, contributing to the multidisciplinary management of this complex condition.

## Introduction

Autism spectrum disorder (ASD) is a multifaceted neurodevelopmental condition characterized by persistent challenges in social communication and interaction, as well as restricted, repetitive patterns of behavior, interests, or activities. According to the Centers for Disease Control and Prevention, ASD affects approximately 1 in 54 children in the United States, highlighting its prevalence and significance as a public health concern [1]. While the etiology of ASD remains complex and multifactorial, it is widely recognized that individuals with ASD often present with a diverse array of symptoms and comorbidities, ranging from sensory sensitivities to gastrointestinal issues [2,3].

Among the myriad of challenges faced by individuals with ASD, speech impairment and attention deficit are common manifestations that can profoundly impact their daily functioning and quality of life. Speech impairment may encompass difficulties in expressive language, articulation, and pragmatic communication skills, making it challenging for individuals with ASD to effectively convey their thoughts, needs, and emotions [4]. Similarly, attention deficit, characterized by difficulties in sustaining attention, controlling impulses, and organizing tasks, can impede learning, social interaction, and academic achievement in children with ASD [5].

Traditional treatment approaches for ASD typically involve a multidisciplinary approach, including behavioral therapy, speech-language therapy, occupational therapy, and pharmacological interventions. However, there is growing interest in exploring complementary and alternative therapies, such as osteopathic manipulation, as adjunctive modalities to address the complex needs of individuals with ASD. Osteopathic manipulation, rooted in the principles of osteopathic medicine, involves hands-on techniques aimed at optimizing the musculoskeletal system, enhancing circulation, and promoting overall well-being [6-9].

In recent years, there has been increasing interest in exploring complementary and alternative therapies to supplement traditional interventions for ASD. One such therapy that has garnered attention is osteopathic manipulation, a hands-on approach rooted in osteopathic medicine principles. Osteopathic manipulation encompasses a diverse range of techniques aimed at optimizing musculoskeletal function, enhancing circulation, and promoting overall well-being [10].

The rationale behind utilizing osteopathic manipulation in individuals with ASD lies in its holistic approach to health and its potential to address underlying physical dysfunctions that may contribute to the presentation of ASD symptoms. While the exact etiology of ASD remains unclear, there is growing evidence to suggest that structural and biomechanical factors may play a role in the pathophysiology of the disorder [11-14]. Osteopathic manipulation seeks to address these factors by identifying and correcting musculoskeletal imbalances, cranial restrictions, and somatic dysfunctions that may impact neurological function and sensory processing. Despite the lack of large-scale randomized controlled trials evaluating the efficacy of osteopathic manipulation in ASD, anecdotal reports and preliminary studies have shown promising outcomes. These include improvements in communication skills, sensory integration, behavioral regulation, and overall quality of life for individuals with ASD. Additionally, osteopathic manipulation may offer a non-invasive and well-tolerated therapeutic option, particularly for individuals who may experience challenges with traditional interventions or medication management [15].

Given the heterogeneous nature of ASD and the individual variability in treatment response, it is crucial to explore complementary therapies such as osteopathic manipulation as part of a comprehensive treatment approach. By addressing physical dysfunctions and promoting optimal musculoskeletal health, osteopathic manipulation has the potential to complement existing therapies and enhance outcomes for individuals with ASD. In this context, the present case report aims to elucidate the effect of osteopathic manipulation in a child diagnosed with ASD, presenting with speech impairment and attention deficit [16-18].

Osteopathy is a form of alternative medicine that emphasizes the physical manipulation of the body’s muscle tissue and bones to promote healing and overall health. Osteopaths believe that the body has the ability to heal itself, and they focus on treating the underlying causes of pain and dysfunction rather than just addressing the symptoms. The practice of osteopathy is based on the principle that the body’s structure and function are interconnected and that disruptions in one part of the body can affect other parts. Osteopaths use a variety of hands-on techniques, such as massage, stretching, and manipulation, to restore balance and alignment to the musculoskeletal system. In many countries, osteopathy is considered a complementary or alternative therapy, and practitioners are required to undergo extensive training and certification. In some places, osteopaths are licensed healthcare professionals who can diagnose and treat patients independently, while in others they may work alongside medical doctors and other healthcare providers. This case report underscores the importance of considering holistic and integrative approaches in the management of ASD and highlights the potential of osteopathic manipulation as a complementary therapeutic modality in this population.

## Case presentation

Prenatal history

Before starting the assessment and treatment, consent was taken from the child’s father. The subject of this case report is a four-year-old male child diagnosed with ASD, presenting with speech impairment and attention deficit. The prenatal history revealed an uneventful pregnancy, with the mother receiving regular prenatal care and reporting no significant complications. Maternal health during pregnancy was stable, with no history of chronic medical conditions or exposure to teratogenic substances. Routine prenatal screenings, including ultrasounds and genetic testing, were within normal limits, with no indications of fetal anomalies or developmental concerns.

Natal history

The child was born at full term via spontaneous vaginal delivery, with a birth weight of 3.4 kg and Apgar scores of 9 and 9 at one and five minutes, respectively. The newborn exhibited appropriate physiological responses and feeding behaviors.

Postnatal history

The child had a supportive family environment, with adequate access to healthcare resources and parental involvement in early intervention programs. There were no reported perinatal complications or neonatal morbidities requiring hospitalization or specialized medical care. The child’s growth and development were monitored closely by healthcare providers, with ongoing assessments of developmental progress and adjustment of therapeutic interventions as needed.

Presentation and diagnosis

At the age of four, the child was evaluated by a multidisciplinary team and received a diagnosis of ASD based on clinical criteria outlined in the Diagnostic and Statistical Manual of Mental Disorders. The diagnostic assessment revealed deficits in social communication, restricted interests, and repetitive behaviors consistent with ASD. Additionally, the child exhibited significant speech impairment, characterized by limited expressive language, articulation difficulties, and pragmatic communication challenges. Attention deficit was also noted, with difficulties in sustaining attention, following instructions, and engaging in structured activities. Developmental milestones were delayed, with the child demonstrating limited social interaction. Early intervention services were initiated to address developmental delays, including speech therapy and behavioral interventions. After diagnosis, the mother brought his child to the Department of Paediatrics Physiotherapy OPD, Acharya Vinoba Bhave Rural Hospital, Sawangi Meghe, Wardha with complaints of speech impairments and attention deficit. The child was assessed (Table 1) and treatment was initiated.

**Table 1:** Clinical examination.

Anthropometric measurement	At birth	At present
Weight	3.4 kg	23 kg
Height	52 cm	102 cm
Chest circumference	35 cm	48 cm
Head circumference	34 cm	48 cm

Clinical investigation

The brainstem evoked response audiometry (BERA) test measures the electrical activity generated by the auditory nerve and brainstem in response to sound stimuli, providing valuable information about the integrity of the auditory pathway. In this case, the normal findings on BERA suggested that the child’s auditory system was functioning within typical parameters, with no evidence of structural or functional abnormalities that could contribute to auditory processing difficulties. While auditory processing difficulties are commonly reported in individuals with ASD, the normal BERA results suggested that other factors may be contributing to the child’s presentation of ASD symptoms, such as social communication challenges, sensory sensitivities, and cognitive differences. It is important to recognize that ASD is a complex neurodevelopmental disorder with heterogeneous manifestations, and the absence of auditory abnormalities on BERA does not rule out the diagnosis of ASD (Table 2).

**Table 2:** Brainstem evoked response audiometry (BERA) test (data represented as N). EP: evoked potentials; SPL: sound pressure level; Cz-M: vertex mastoid; R: right; L: left

N	Rec.sites	I Lat., ms	Ia Lat., ms	II Lat., ms	III Lat., ms	IIIa Lat., ms	IV Lat., ms	V Lat., ms	I-III Lat., ms	III-V Lat., ms	I-V Lat., ms	Stim.side	Stimulus
1	Cz-M	1.43	1.75	3.25	NA	NA	5.15	6.63	NA	NA	5.2	R	±110 dB SPL
2	Cz-M	1.53	2.85	3.35	4.6	4.85	5.25	6.68	3.08	2.08	5.15	R	±100 dB SPL
3	Cz-M	2.38	2.78	3.5	NA	4.6	5.4	NA	NA	NA	NA	R	±90 dB SPL
4	Cz-M	2.35	3.23	3.55	3.95	4.75	5.5	5.9	1.6	1.95	3.55	R	±80 dB SPL
5	Cz-M	1.73	2.45	2.8	3.75	4.03	4.3	5.83	2.03	2.08	4.1	R	±70 dB SPL
6	Cz-M	1.83	2.65	2.88	3.95	5.15	5.35	5.95	2.13	2.0	4.13	R	±60 dB SPL
7	Cz-M	2.2	NA	NA	3.75	4.35	5.68	6.63	1.55	2.88	4.43	R	±50 dB SPL
8	Cz-M	1.93	NA	NA	3.93	4.65	4.88	5.53	2.0	1.6	3.6	R	±40 dB SPL
9	Cz-M	1.35	2.83	3.3	4.4	4.68	5.25	6.78	3.05	2.38	5.43	L	±110 dB SPL
10	Cz-M	1.53	2.9	3.35	NA	NA	5.2	6.8	NA	NA	5.28	L	±100 dB SPL
11	Cz-M	1.6	2.88	3.5	4.65	4.83	5.38	NA	3.05	NA	NA	L	±90 dB SPL
12	Cz-M	2.15	3.0	3.53	4.6	4.93	5.4	5.73	2.45	1.13	3.58	L	±80 dB SPL
13	Cz-M	2.3	2.88	NA	3.9	4.8	5.33	5.78	1.6	1.88	3.48	L	±60 dB SPL
14	Cz-M	2.08	3.1	NA	NA	4.3	5.0	6.05	NA	NA	3.98	L	±40 dB SPL
15	Cz-M	1.88	3.28	3.6	NA	4.63	6.08	6.53	NA	NA	4.65	L	±50 dB SPL

Intervention

The child underwent osteopathic manipulation sessions over a period of 12 weeks, focusing on addressing identified somatic dysfunctions and optimizing musculoskeletal function. Treatment goals aimed to improve cranial mobility, release tension in soft tissues, and restore optimal biomechanical alignment to facilitate neurological integration and sensory processing (Table 3).

**Table 3:** Osteopathic manipulation.

Technique	Procedure	Intensity
Suboccipital release technique	The individual is positioned on their back while the therapist sits at the head of the person. The therapist then places the tips of their fingers on the muscles at the base of the skull and applies gentle pressure in an anterior direction. Gravity assists in the process, gradually causing the muscles to relax and soften. The therapist maintains this pressure until the muscle tissue noticeably relaxes	The procedure can be repeated two to three times depending on the patient’s sensitivity
Sternocleidomastoid fascial release	The individual lies on their back, while the therapist stands at the level of the rib cage’s costal arch. The therapist then places both hands on the costal arch and begins to gently move the diaphragm in a figure-of-eight pattern. This movement should be synchronized with the individual’s breathing rhythm and performed smoothly to ensure that the patient feels no discomfort	The procedure can be repeated two to three times depending on the tonicity of the muscle
Diaphragm mobilization	The individual is positioned on their back, while the therapist stands at the level of the rib cage’s costal arch. The therapist then places both hands on the costal arch and initiates a figure-of-eight motion to mobilize the diaphragm. This should be performed rhythmically in sync with the individual’s breathing, ensuring that the patient remains comfortable throughout the procedure	The procedure can be repeated two to three times depending on the mobility of the diaphragm
End to end stretch of the descending colon	The individual is positioned lying on their side, facing toward the right, with their legs bent. The therapist stands behind the individual, placing their right hand under the right costal arch, curling the fingers in a posterior and upward direction, and then laterally toward the right colic flexure. Simultaneously, with the left hand, the therapist grasps and holds the beginning of the ascending colon, located at the level of the iliac crest, stabilizing it posteriorly. The therapist then applies force with the right hand in a posterior and lateral direction, while the left hand applies inferior force, resulting in a lengthwise stretch of the descending colon	The procedure can be repeated two to three times depending on the patient’s sensitivity
End to end stretch of the ascending colon	The individual is positioned lying on their side, facing toward the left, with their legs bent. The therapist stands behind the individual, using their left hand to curl the fingers under the right costal arch, directing them posteriorly and laterally toward the right colic flexure. Simultaneously, with the right hand, the therapist grasps and holds the beginning of the ascending colon, located at the level of the iliac crest, stabilizing it posteriorly. The therapist then applies force with the left hand in a posterior and lateral direction, while the right hand applies inferior force, resulting in a lengthwise stretch of the ascending colon	The procedure can be repeated two to three times depending on the patient’s sensitivity
Peritoneum grasping technique	The individual lies on their back with bent legs. The therapist positions themselves at the level of the abdomen and, using both hands, assesses the tone and tension in the abdominal area. Once the targeted tissue is identified, it is grasped and pulled anteriorly, holding the grasp for 30 seconds to 1 minute. The grasp should extend beyond just the abdominal wall level, reaching the peritoneum	The procedure can be repeated two to three times depending on the patient’s sensitivity. The technique should be applied with caution as it is very painful but can be repeated two times according to the patient’s response

Outcome measures

WeeFIM (Functional Independence Measure for Children) is a standardized assessment tool designed to evaluate the functional independence of children in activities of daily living (ADLs). It consists of six domains, namely, self-care, sphincter control, transfers, locomotion, communication, and social cognition. Each domain is scored on a scale from 1 to 7, with higher scores indicating greater independence. The WeeFIM provides valuable information about the child’s level of functional ability and the need for assistance in various areas of daily life. The Corners Rating Scale is a behavioral assessment tool used to evaluate the severity of autism symptoms. It consists of items assessing social interaction, communication, and repetitive behaviors, with ratings ranging from 0 (no impairment) to 4 (severe impairment). The Corners Rating Scale provides quantitative data on the severity of autism symptoms, allowing for tracking of symptom progression over time and evaluation of treatment effectiveness.

The Attention Control Scale is a self-report measure designed to assess an individual’s ability to control their attention and focus. It consists of items assessing attentional control, distractibility, and ability to maintain concentration in various situations. Higher scores indicate greater attentional control and ability to sustain focus. The Attention Control Scale provides insight into the child’s attentional abilities, which are often impaired in individuals with ASD. The Indian Scale for Assessment of Autism (ISAA) is a culturally adapted tool for assessing ASD symptoms in Indian children. It consists of items assessing social interaction, communication, and restricted and repetitive behaviors, with ratings based on observations and caregiver reports. The ISAA provides a comprehensive assessment of autism symptoms tailored to the Indian context, allowing for accurate diagnosis and treatment planning in this population (Table 4).

**Table 4:** Outcome measures. Corners rating scale: 0 (no impairment) to 4 (severe impairment); Attention Control Scale: 4 (no impairment) to 0 (severe impairment); Indian Scale for Assessment of Autism: less than 70 normal, 70 to 106 mild autism, 107 to 153 moderate autism, and more than 153 severe autism.

	Pretreatment	Follow-up (12 weeks)
WeeFIM	Score 2	Score 5
Corners Rating Scale	Score 4	Score 1
Attention Control Scale	Score 1	Score 4
Indian Scale for Assessment of Autism (ISAA)	140	70

## Discussion

The case report highlights significant improvements in speech fluency and attention span following the implementation of osteopathic manipulation techniques. The suboccipital release technique, aimed at releasing tension in the suboccipital muscles and improving cranial mobility, facilitated enhanced neurological integration and communication pathways, leading to improvements in speech. Similarly, sternocleidomastoid fascial release and diaphragm mobilization may have contributed to improved respiratory function and vocalization, thereby enhancing speech production. The observed enhancements in attention span could be attributed to the calming effect of osteopathic manipulation techniques, promoting relaxation and reducing sensory overload, which are common challenges in individuals with ASD.

The mechanisms underlying the observed improvements in speech impairment and attention deficit following osteopathic manipulation warrant further investigation. It is hypothesized that these techniques influence neurophysiological processes, such as modulation of the autonomic nervous system, release of endorphins, and regulation of sensory processing, thereby promoting improved communication skills and attention regulation. Additionally, osteopathic manipulation addresses musculoskeletal dysfunctions and cranial restrictions that contribute to speech impairments and attention deficits in individuals with ASD.

It is essential to recognize that ASD is a complex neurodevelopmental disorder with multifactorial etiology and heterogeneous presentation. While osteopathic manipulation offers benefits in addressing specific symptoms such as speech impairment and attention deficit, it is unlikely to provide a comprehensive solution for all aspects of ASD. Therefore, a multidisciplinary approach that integrates osteopathic manipulation with other evidence-based interventions, such as speech therapy, behavioral interventions, and educational support, is necessary to address the diverse needs of individuals with ASD [19,20].

The case report has several limitations, including its observational nature. Additionally, the generalizability of findings may be limited by the unique characteristics of the individual case and variations in response to osteopathic manipulation across different individuals with ASD. Future research should employ rigorous study designs, larger sample sizes, and objective outcome measures to validate the efficacy of osteopathic manipulation in ASD.

Despite the limitations, the case report underscores the potential of osteopathic manipulation as an adjunctive therapy for addressing speech impairment and attention deficit in individuals with ASD. Further research is warranted to elucidate the specific mechanisms of action, optimal treatment protocols, and long-term effects of osteopathic manipulation in ASD. Additionally, collaborative efforts between osteopathic physicians, speech-language pathologists, and other healthcare professionals are essential for integrating osteopathic care into comprehensive treatment plans for individuals with ASD.

## Conclusions

This case report provides valuable insights into the potential benefits of osteopathic manipulation in improving speech impairment and attention deficit in a child with ASD. While preliminary findings are promising, further research is needed to establish the efficacy, mechanisms of action, and clinical utility of osteopathic manipulation as a therapeutic intervention for individuals with ASD.
